# Response of pulmonary artery intimal sarcoma to surgery, radiotherapy and chemotherapy: a case report

**DOI:** 10.1186/1752-1947-2-217

**Published:** 2008-06-25

**Authors:** Hong-qing Long, Qin Qin, Cong-hua Xie

**Affiliations:** 1 Medical Department of Xianning University, Xianning, Hubei 437000, PR China; 2 Department of Cancer Radio-chemotherapy, Zhongnan Hospital and Cancer Center of Wuhan University, Donghu Road, Wuhan, Hubei 430071, PR China

## Abstract

**Introduction:**

Pulmonary artery intimal sarcoma is a rare disease with no characteristic symptoms. It is difficult to diagnose early and is frequently misdiagnosed as a pulmonary embolism.

**Case presentation:**

Here we report a case of pulmonary artery intimal sarcoma in a 54-year-old woman presenting with complaints of shortness of breath on exertion. Echocardiography and a computed tomography scan showed that the right pulmonary artery trunk was blocked by a low-density mass. The patient was diagnosed with pulmonary artery intimal sarcoma by pathology and a complete mass resection was performed. After experiencing 10 months of disease-free survival, she was re-admitted because of the recurrence and metastasis of the tumor. Radiotherapy and chemotherapy were performed; however, only limited success was achieved. The patient died 15 months after the initial onset of symptoms.

**Conclusion:**

Some patients with intimal sarcoma of the pulmonary artery can benefit from radiotherapy and chemotherapy as well as surgery.

## Introduction

Intimal sarcoma of the pulmonary artery (PA) is a very rare tumor with poor prognosis. It is frequently misdiagnosed as pulmonary thromboembolism, and in most cases the definitive diagnosis is made during surgery or upon autopsy. The defining feature of this sarcoma is local growth with slight ability to metastasize [1]. Here we present and discuss a case of PA intimal sarcoma with extensive metastases 10 months after the initial diagnosis was made during surgery.

## Case presentation

**Figure 1:**
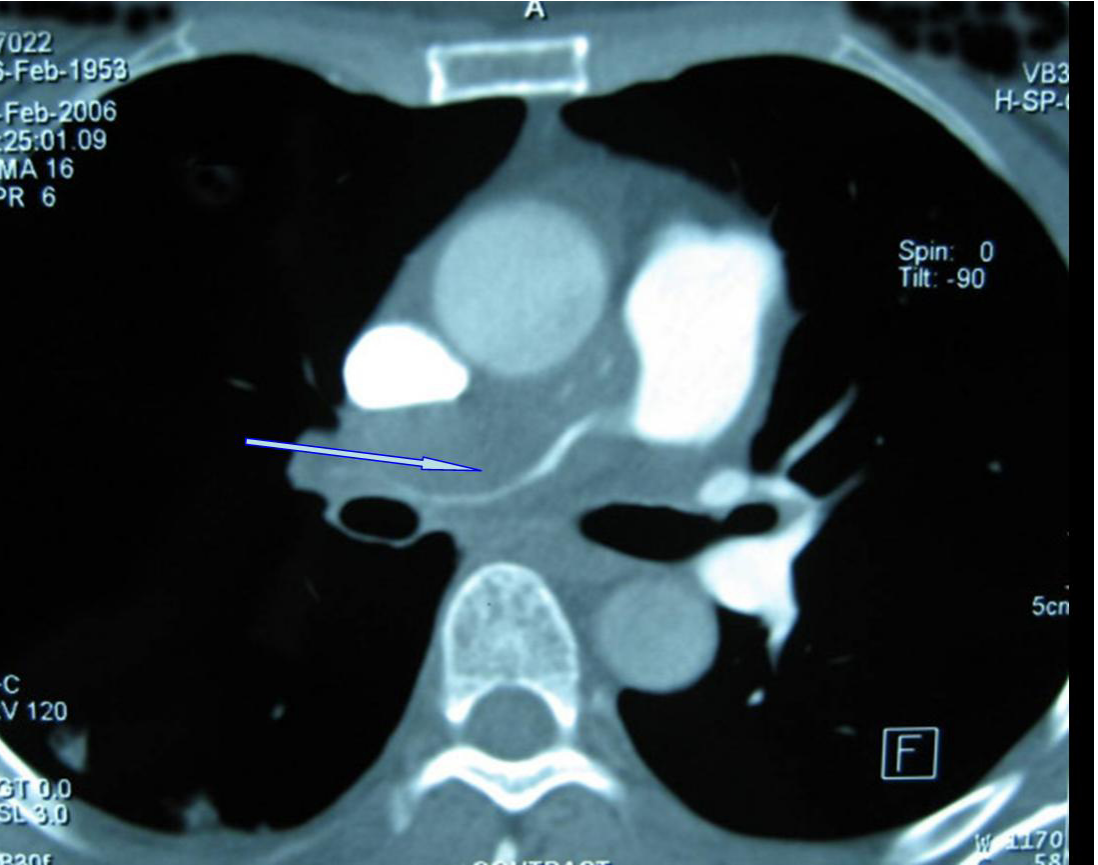
A computed tomography scan of the chest shows the main pulmonary artery was obstructed circumferentially.

The patient, a 54-year-old woman, presented with shortness of breath on exertion over the previous three months. Physical examination revealed slight jugular vein distention (JVD); a grade III to VI systolic murmur, which was heard at the tricuspid area; and mild edema of both legs. Laboratory reports were within normal ranges. Echocardiography showed that the right PA (RPA) was almost completely obstructed by a low echogenic mass, and the left PA (LPA) was also partially obstructed, and the superior vena cava (SVC) was dilated with a diameter of about 23 mm. Right ventricle enlargement and tricuspid insufficiency were also revealed. A helical computed tomography (CT) scan showed that the RPA trunk was blocked by a soft tissue mass which grew circumferentially, with only line-like contrast passing through it (Figure 1). The LPA was thickened interiorly, with favorable contrast perfusion to its branches. On the basis of these findings, the patient was diagnosed with PA thromboembolism.

**Figure 2:**
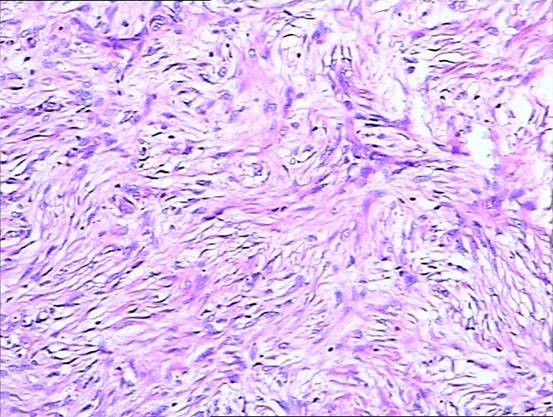
**Pathological findings of surgical specimen show abundant spindle cells**. Hematoxylin and eosin stain ×100.

During surgery, a local soft mass measuring approximately 1.5 × 1.8 × 4.0 cm^3 ^was completely resected from the RPA. The diagnosis of intimal sarcoma was made by pathological examination (Figure 2). Atypical spindle cells were observed. Immunohistochemical analysis was positive for vimentin and weakly positive for CD34, while CD117, S-100, smooth muscle actin, desmin, and CD68 were negative. Following surgery the symptoms were relieved, and echocardiography showed normal blood flow in the PA trunk, RPA and LPA as well as in their proximal branches. The patient was then discharged.

**Figure 3:**
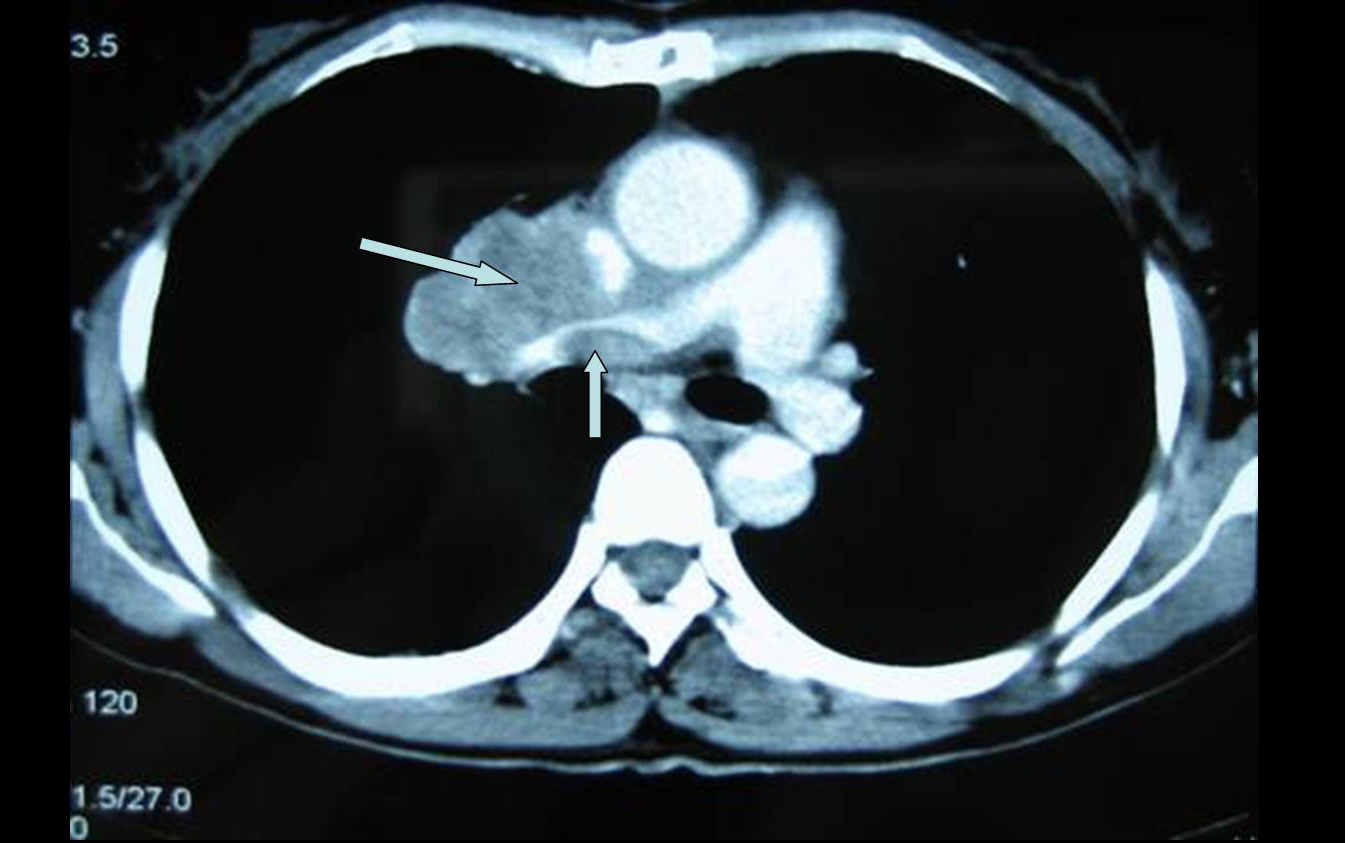
A computed tomography scan of the chest shows the recurrence of pulmonary artery intimal sarcoma.

Ten months later, the patient was referred to our department with complaints of shortness of breath on exertion, facial swelling and pain in the right upper quadrant. On physical examination no abnormalities were found except for facial swelling and JVD. Helical CT scan revealed a solid mass in the right pulmonary hilum, with no clear boundary to the mediastinum. The mass could be enhanced asymmetrically, with a central low-density necrosis invading the SVC and RPA trunk (Figure 3). In addition, a low-density cycloid mass with a clear boundary was detected on the quadrate lobe of the liver. Based on these findings, a diagnosis of a local recurrence of intimal sarcoma of the PA was made, with suspected metastasis to the liver. Three-dimensional conformal radiation therapy (3D-CRT) to the mediastinum was performed with a total dose of 60 Gy using 15 MV X-ray in 30 fractions. Dyspnea and facial swelling were relieved.

Thirteen days after radiotherapy, the patient complained of pain in the right upper quadrant, loss of appetite, nausea and vomiting. An enlarged metastatic mass in the liver was detected by CT. Transcatheter Arterial Chemoembolization (TACE) was performed on the mass in the liver. After treatment, the symptoms were relieved. Two months later, a further CT scan showed extensive metastases to lung, liver and both adrenal glands. The patient died 2 weeks later.

## Discussion

Intimal sarcoma is a malignant mesenchymal tumor that arises in large vessels including the aorta and PA. These tumors are characterised by intraluminal growth with obstruction of the tract and seeding of emboli [1,2]. Intimal sarcoma is a rare tumor, and the incidence of PA intimal sarcoma is almost twice that of sarcomas of aortic origin. It mainly occurs in adults within the age range of 13 to 86 years, with a female predominance (the female to male ratio is 1.3:1). There is no gender variation in the incidence of aortic intimal sarcoma. The mean age of patients diagnosed with PA intimal sarcoma is 48 years, while the mean age of patients at diagnosis of aortic intimal sarcoma is 62 years [3,4].

PA intimal sarcoma arises from the intimal layer of the right, left and main PA. In rare cases, it extends in a retrograde manner to the pulmonary valve and right ventricle [5]. Approximately 40% of patients develop a direct invasion or metastasis to the lung, while systematic spread to the kidneys, brain, or adrenal glands occurs in about 20% of cases.

Patients with intimal sarcoma present with various symptoms. The most common initial symptoms are dyspnea, hemoptysis and chest pain [1]. Due to the rarity and insidious growth of intimal sarcoma, diagnosis is always delayed or made at surgery or autopsy. PA intimal sarcoma is often misdiagnosed as pulmonary thromboembolism.

Surgery offers the best way to prolong survival and is successful only if complete resection of tumor is performed [6]. Postoperative chemotherapy has been reported to be effective in some cases [2,7], but its role in the treatment of PA intimal sarcoma is still not clearly defined. The same is true for radiation therapy and postoperative anticoagulation therapy [8]. The prognosis of PA intimal sarcoma is poor, and survival is usually 12 to 18 months [2,3].

In this case, the patient had a 10-month symptom-free period after surgery, even though the surgery was a simple local mass resection, confirming the positive role of surgery in the management of PA intimal sarcoma. When tumor relapse occurred in the pulmonary hilum, local 3D-CRT to the mediastinum successfully controlled symptoms of dyspnea and facial swelling. This suggests that intimal sarcoma of the PA may be sensitive to radiation in some cases. In addition, TACE of a metastasis in the liver also relieved pain for 2 months. Unfortunately, further systemic metastases finally developed, and the total survival time for this patient was approximately 15 months from the onset of symptoms.

## Conclusion

PA intimal sarcoma is a rare tumor. Surgery can prolong the survival of patients and therapies such as chemotherapy and radiotherapy may also contribute to the management of this disease, and where appropriate should be recommended. In addition, systematic evaluation of possible metastasis should be considered as this tumor has the capacity to metastasize.

## Abbreviations

3D-CRT: three-dimensional conformal radiation therapy; CT: computed tomography; JVD: jugular vein distention; LPA: left pulmonary artery; PA: pulmonary artery; RPA: right pulmonary artery; SVC: superior vena cava; TACE: transcatheter arterial chemoembolization.

## Consent

Written informed consent was obtained from the patient's next-of-kin for publication of this case report and accompanying images. A copy of the written consent is available for review by the Editor-in-Chief of this journal.

## Competing interests

The authors declare that they have no competing interests.

## Authors' contributions

HqL collected the data, QQ drafted the manuscript, and ChX revised and approved the final manuscript. All authors read and approved the final manuscript.
